# A Chimeric 18L1-45RG1 Virus-Like Particle Vaccine Cross-Protects against Oncogenic Alpha-7 Human Papillomavirus Types

**DOI:** 10.1371/journal.pone.0120152

**Published:** 2015-03-19

**Authors:** Bettina Huber, Christina Schellenbacher, Christoph Jindra, Dieter Fink, Saeed Shafti-Keramat, Reinhard Kirnbauer

**Affiliations:** 1 Department of Dermatology, Division of Immunology, Allergy and Infectious Diseases (DIAID), Laboratory of Viral Oncology (LVO), Medical University Vienna (MUW), Vienna, Austria; 2 Institute of Laboratory Animal Science, Department of Biomedical Science, Veterinary University Vienna, Vienna, Austria; University of Nebraska-Lincoln, UNITED STATES

## Abstract

Persistent infection with oncogenic human papillomaviruses (HPV) types causes all cervical and a subset of other anogenital and oropharyngeal carcinomas. Four high-risk (hr) mucosal types HPV16, 18, 45, or 59 cause almost all cervical adenocarcinomas (AC), a subset of cervical cancer (CxC). Although the incidence of cervical squamous cell carcinoma (SCC) has dramatically decreased following introduction of Papanicolaou (PAP) screening, the proportion of AC has relatively increased. Cervical SCC arise mainly from the ectocervix, whereas AC originate primarily from the endocervical canal, which is less accessible to obtain viable PAP smears. Licensed (bivalent and quadrivalent) HPV vaccines comprise virus-like particles (VLP) of the most important hr HPV16 and 18, self-assembled from the major capsid protein L1. Due to mainly type-restricted efficacy, both vaccines do not target 13 additional hr mucosal types causing 30% of CxC. The papillomavirus genus alpha species 7 (α7) includes a group of hr types of which HPV18, 45, 59 are proportionally overrepresented in cervical AC and only partially (HPV18) targeted by current vaccines. To target these types, we generated a chimeric vaccine antigen that consists of a cross-neutralizing epitope (homologue of HPV16 RG1) of the L2 minor capsid protein of HPV45 genetically inserted into a surface loop of HPV18 L1 VLP (18L1-45RG1). Vaccination of NZW rabbits with 18L1-45RG1 VLP plus alum-MPL adjuvant induced high-titer neutralizing antibodies against homologous HPV18, that cross-neutralized non-cognate hr α7 types HPV39, 45, 68, but not HPV59, and low risk HPV70 in vitro, and induced a robust L1-specific cellular immune response. Passive immunization protected mice against experimental vaginal challenge with pseudovirions of HPV18, 39, 45 and 68, but not HPV59 or the distantly related α9 type HPV16. 18L1-45RG1 VLP might be combined with our previously described 16L1-16RG1 VLP to develop a second generation bivalent vaccine with extended spectrum against hr HPV.

## Introduction

High-risk (hr) mucosal human papillomaviruses (HPV) are the causative agents of practically all cervical cancers (CxC) worldwide. Altogether, there are more than 15 hr mucosal HPV types known so far, the majority belonging to species α9 (HPV16, 31, 33, 35, 52, 58) and α7 (HPV18, 39, 45, 59, 68), and in addition to α5 (HPV51), α6 (both HPV56, 66), and α11 (HPV73) [1,2]. HPV16 is the most important hr type causing squamous cell cancers (SCC) arising from the cervical transformation zone, and HPV18 is preferentially associated with adenocarcinoma (AC) of the endocervix, although both types can cause cancers of both epithelial sites. Together, HPV16, 18 and 45 are responsible for about 77% of all CxC [3], and 94% of the subset of cervical AC cases worldwide. About 10% of the worldwide population is infected with HPV at the ano-genital site [4]. Genital HPV is the most common sexually transmitted infection (STI), with a lifetime cumulative risk of 70%.

The two licensed HPV vaccines (bivalent Cervarix and quadrivalent Gardasil) contain virus-like particles (VLP) self-assembled from recombinant L1 major capsid proteins of hr HPV16 or 18. Gardasil additionally contains VLP of low-risk (lr) HPV6 and 11 that cause 90% of benign ano-genital warts. Both prophylactic vaccines provide mainly type-restricted protection against the included vaccine types, but only limited cross-protection against types HPV31, 33 (closely related to HPV16) and HPV45 (closely related to HPV18). Thus, cervical screening cannot be abandoned in vaccinated women. Also, high costs of current vaccines and poor infrastructure limit access for both primary (vaccination) and secondary (Pap screening) prevention in developing countries that carry more than 80% of the CxC burden worldwide.

To target the majority of hr mucosal HPV a nonavalent VLP vaccine is in clinical trials. However, the complexity of such a vaccine is unlikely to reduce costs of manufacturing. Alternative strategies for the development of broad-spectrum second generation vaccines have focused on the minor structural protein L2 of papillomaviruses. The N-terminus of L2 contains type-common epitopes that can induce cross-neutralizing antibodies of relatively low titer that efficiently cross-protect in vivo [5–14]. Several studies have attempted to enhance L2’s antigenicity, by either fusion of L2 proteins of multiple HPV types and/or L2 concatemers [6,15], or multimeric presentation of L2 cross-neutralizing epitopes (e.g. HPV16 L2 amino-acid (aa) 69–81, 108–120 or 56–75) by a VLP scaffold of bovine papillomavirus 1 (BPV1) [11], HPV16 [16], or bacteriophages [9].

Other approaches are based upon the L2 cross-neutralization B cell epitope called RG1, which refers to a 20 aa (17–36) N-terminal peptide of the HPV16 L2 protein that is highly conserved among many PV types. We have previously generated chimeric VLP, by genetic insertion of the RG1 epitope of HPV16 L2 into the DE surface loop of HPV16 L1, as a single-formulation vaccine approach [8,17–19]. Expression as a fusion protein resulted in self-assembly into ‘RG1-VLP’ (16L1–16RG1) that repetitively display the RG1 peptide on the capsid surface. Immunizations induced broadly cross-neutralizing antisera against even distantly related HPV types in vitro. In an experimental mouse model, passive serum transfer conferred efficient cross-protection against vaginal challenge with pseudovirions (PsV) of a panel of heterologous HPV types including hr and lr mucosal types. Therefore, RG1-VLP is a promising approach for the development of broad-spectrum vaccines to target the large number of medically important HPV types.

There are two main CxC types, SCC and AC, and less common types like adenosquamous, clear-cell or small-cell carcinomas. Since the establishment of cervical PAP smear screening programs the incidence of CxC cases has decreased >80% in more than 13 developed countries [20]. However, incidences of AC proportionally increased relative to SCC cases, especially in women under the age of 40, probably because detection of AC arising from the endocervical canal by cytological screening is less efficient than detection of SCC [21]. In the 1950s-60s, SCC accounted for 95% of all CxC cases in the US, whereas AC and adenosquamous cell carcinoma accounted for only 5% [22]. Since then, SCC cases declined to 75–80%, while the proportion of AC and adenosquamous carcinoma cases increased to 20–25%.

Almost all cervical SCC have been shown to contain HPV DNA, whereas previous studies reported variable HPV prevalences in AC [23]. More recently however, it has been firmly established that similar to SCC, AC is also invariably HPV-associated (except non-mucinous cases), and previously reported differences in prevalence might have arisen due to technical factors related to sampling, viral DNA detection methods, or histologic misclassification. In cervical carcinogenesis the viral DNA, in particular of types HPV16, 18, or 45, becomes progressively integrated into the cellular chromosome with the E2 and L1 genes deleted, the latter of which is often used as target for PCR-based detection tests [24]. Reanalysis of former as HPV-negative diagnosed CxC cases revealed an HPV prevalence in invasive CxC worldwide of 99%, and the confirmed rarity of HPV-negative CxC [23]. Studies of genotype distributions in all types of CxC worldwide have shown that HPV16 accounts for about 53–59%, HPV18 for 13–18%, and HPV45 for 6–7% of cases. In cervical precursor lesions HPV16 (56%) is the most important type as well, whereas HPV types 31 and 33 (12% and 8%, respectively) play a more prominent role [25,26]. In cervical AC HPV16 is the predominant type (52%), followed by HPV18 (39%) and HPV45 (6.2%). In countries from Southeast Asia, HPV18 is even more prevalent in AC (>50%) than HPV16 (>30%) or HPV45 (<10%), whereas HPV16 is also most prevalent in SCC [27]. HPV infection is the most important risk factor for cervical AC, associated with an 80-fold increased risk [26] and further risk factors include low level of educational attainment, poor education, first intercourse at a young age, a large number of sexual contacts, history of sexually transmitted infections (STI) and practicing anal intercourse.

As current screening programs less efficiently detect AC, and licensed vaccines only provide limited protection, a vaccine targeting the majority of hr types, in particular those causing cervical AC, is highly desirable. In the present study we generated a chimeric L1-RG1 VLP vaccine that specifically targets the α7 group of HPV, which is overrepresented in AC, intending to induce long-lasting and cross-protective immune responses.

## Material and Methods

### Cell lines

*Spodoptera frugiperda* (Sf9) insect cells were kept in Grace’s media supplemented with 5% fetal calf serum (FCS) and 0.5% Pluronics (Gibco) at 27°C. 293TT cells were grown in DMEM, 10% FCS, 1% non-essential amino acid (NEAA) and 400 μg/mL Hygromycin B (Invitrogen). PGSA-745 and CHOΔfurin cells (kindly provided by Patricia Day, NCI) were kept in DMEM, 10% FCS, 1% Antibiotic-Antimycotic (Gibco) and 10mM proline (Sigma). HaCaT cells were propagated in DMEM, 10% FCS.

### Pseudovirions (PsV)

PsV were produced in 293TT cells as described [28,29]. Briefly, 1.5x10^7^ 293TT cells (175cm^2^ flask) were plated and one day later transfected with 38μg HPV L1 and L2 plasmid(s) and 38μg reporter plasmid. 48 hours later, cells were lysed in PBS with 9.5mM MgCl_2_, 0.5% (*v/v*) Triton-X100, 0.25% Ammonium sulfate, 0,01% benzonase (Novagen) and 0,01% Plasmid Safe (Epicentre). For maturation cell lysates were incubated 24 hours at 37°C. Finally, PsV were salt extracted and purified by ultracentrifugation on an Optiprep step gradient (27%, 33%, 39%) (Sigma). Plasmids for codon-optimized L1 and L2 expression of HPV16 (p16shell) and HPV18 (peL1fB and peL2bhb) were provided by J. Schiller, NCI, for HPV45 (p45shell) by J. Dillner, Karolinska Institute, and for HPV39, HPV59, HPV68, HPV70 (all in pVITRO) by R. Roden, Johns Hopkins University.

The reporter plasmid pYSEAP (encoding secreted alkaline phosphatase) was used for in vitro L1 or L2-based PsV assays, and pCLUC (encoding luciferase) for in vitro L2-based PsV assays and in vivo experiments.

### Baculovirus expression of chimeric L1-RG1 proteins

The chimeric HPV18L1–45RG1 fusion construct was designed using CLC DNA workbench based on HPV18 L1 (accession number HPV18 AY262282) and HPV45 L2 (X74479), codon optimized for mammalian expression and synthesized by GeneArt (Regensburg, Germany). The gene sequence encoding the 20 aa (residues 16–35) RG1 epitope of HPV45 was inserted into the DE loop of HPV18 L1 between aa positions 134/135. The purified (Qiagen’s Gel extraction kit) L1-RG1 fusion gene was subcloned into the baculovirus transfer vector pSynwtVI^-^ using BglII and KpnI sites [30]. Recombinant vectors were verified by bidirectional sequencing (VBC-Biotech, Vienna, Austria).

Transfer vector and linearized baculovirus DNA (BaculoGold; BD Bioscience) were co-transfected into Sf9 cells and recombinant baculoviruses isolated by plaque assay. Following expression in insect cells, VLP were purified as described [30–32]. Briefly, Sf9 insect cells were infected with amplified high-titer baculovirus stocks for three days. Cells were harvested and lysed by sonication in a breaking buffer (PBS + 0.8M NaCl + 2mM CaCl_2_ + 1mM phenylmethylsulfonyl fluoride (PMSF)) and incubated overnight with addition of 0.5% Brij58. VLP were purified by ultracentrifugation on sucrose-PBS cushions [35% (*w/v*)] and CsCl-PBS [29% (*w/w*)] density gradients.

### Transmission electron microscopy (TEM)

Assembly of chimeric HPV18L1–45RG1 fusion proteins into complete 50–60nm VLP structures was verified by transmission electron microscopy (TEM) using a JEOL 1010 electron microscope at 80kV. Briefly, purified VLP were loaded onto glow-discharged carbon-copper grids, fixed with 2.5% glutaraldehyde and negatively stained with 1% uranylacetate. Micrographs were taken at a 30.000x magnification.

### Western blotting

Sf9 cells were infected with recombinant baculovirus for three days, harvested, and cell-lysates or purified protein were analyzed by SDS electrophoresis and Coomassie staining, or Western blotting using Camvir-1 (1:10,000 dilution; Abcam, Cambridge, UK), or antiserum to HPV16 L2 aa 11–200 (1:5,000 dilution; R. Roden, Johns Hopkins) and a goat anti-mouse IgG-HRP (horseradish peroxidase) coupled secondary antibody (Biorad) to verify the recombinant protein.

### Enzyme-linked immunosorbent assay (ELISA)

Antigenicity of chimeric 18L1–45RG1 and wild type (wt) HPV18L1 VLP was analyzed by native ELISA. In short, 0.1μg native VLP in 100μl PBS were attached onto Maxisorp 96-well plates (Nunc) overnight at 4°C. The next day wells were washed, blocked by 0.5% milk/PBS, and antibody serial dilutions (ranging from 1:200 to 1:204,800) added in triplicates for one hour. Monoclonal antibodies (mAb) used were HPV18-specific conformation-dependent H18.G10 and H18.J4 (N. Christensen, Hershey), or rabbit antiserum raised against HPV16 L2 aa 11–200 (R. Roden, Johns Hopkins), or HPV16-raised mAb Camvir-1 (BD Biosciences), the latter directed against a linear epitope shared by many papillomavirus types including HPV18 L1. The anti-IgG (H+L) HRP-coupled second antibody (1:10,000 dilution; BioRad) was added and incubated for 45 minutes, prior to addition of substrate (2,2'-azino-bis(3-ethylbenzothiazoline-6-sulphonic acid); Roche), and the OD at 405nm determined with an ELISA reader (Opsys MR, Dynex Technologies). For denaturing ELISA, chimeric VLP were diluted into denaturation buffer (0.2 M NaHCO3 pH 10.6 plus freshly added 0.01 M DTT), added at 0.1μg / well, dried onto the plate at 37°C overnight (lid taken off) and wells washed 3x with PBS prior to blocking with 0.5% milk/PBS and addition of serial dilutions of Camvir-1 or rabbit antiserum to HPV16 L2.

Immune sera raised in NZW rabbits were analyzed by ELISA using a HPV45RG1 biotinylated peptide (JPT peptide technologies GmbH, Germany). Briefly, 1μg RG1 peptide in 100μL coating buffer (1M Tris HCl pH 7,4, 1M NaCl, 0,001% Tween-20) were attached overnight to Streptavidin plates (Nunc immobilizer Streptavidin F96 clear) at 4°C. Wells were washed and incubated with coating buffer for 5 minutes and blocked with 1% milk-PBS overnight at 4°C. On the next day, wells were again washed with PBS and incubated in triplicates with serial dilutions of sera raised against either HPV18L1–45RG1 VLP, HPV18L1 VLP or pre-immune sera (dilutions ranging from 1:200 to 1:51,200) for 1 hour at room temperature. Following PBS washing, 2^nd^ antibody goat-anti IgG-rabbit HRP (1:10,000 dilution; BioRad) was added for 45 minutes at room temperature using an ELISA plate shaker, prior addition of substrate (2,2'-azino-bis(3-ethylbenzothiazoline-6-sulphonic acid); Roche), and the OD at 405nm determined with an ELISA reader (Opsys MR, Dynex Technologies). ELISA titers are reported positive for values greater than the mean OD of pre-immune sera plus 3 standard deviation values (SD).

### Immunizations

Gradient-purified protein was extensively dialyzed against PBS, 0.5M NaCl, 1mM CaCl_2_, and 0.02% Tween 80. HPV18L1–45RG1 VLP, 50μg per dose, adjuvanted with 500μg aluminum hydroxide (alum) plus 50μg of the Toll-like receptor 4 agonist 3-O-desacyl-4’-monophosphoryl lipid A (MPL; Sigma), were used to immunize two New Zealand White rabbits (NZW, Charles River Laboratories, Germany) each in a 5-dose regimen (week 0–4–6–8–16) and sera were drawn two weeks after the final boost.

### L1-based pseudovirion (PSV) neutralization assay

The assay was performed as previously described [28]. Briefly, 3x10^4^ 293TT cells per well were plated into a 96-well plate and infected with SEAP-containing PsV that were pre-incubated on ice for one hour with or without (pre-)immune sera. Infection was assessed three days later by measuring SEAP activity in the culture supernatant at 405nm (Opsys MR, Dynex Technologies).

### L2-based PsV neutralization assay

The assay was performed according to Day et al [33]. Briefly, 2*10^6^ HaCaT cells were plated onto 96-well plates for 24 hours at 37°C. Cell media was then removed, cells washed twice with PBS and lysed using PBS, 0.5% Triton X-100, 20mM NH_4_OH for 5 minutes at 37°C. Following lysis, 100μL PBS were added and removed again for three times to gentle wash the remaining extracellular matrix (ECM). PsV were added in CHOΔfurin conditioned medium containing 5^μg^/_ml_ Heparin (Gilvasan Pharma) for 120^μl^/_well_, and incubated overnight at 37°C. ECM was then washed twice with PBS to remove non-attached PsV and antibody serial dilutions added. Control sera raised against HPV18 L1 were kindly provided by Martin Müller, German Cancer Research Center Heidelberg, and John Schiller, NCI. Antibodies were incubated at 37°C for 4–6 hours followed by addition of 8*10^3^ PGSA-745 cells directly to the wells (50^μl^/_well_) and incubation at 37° for two days. For luciferase encoding PsV, assays were evaluated using the Luciferase Assay System (Promega #E1501) and the Cell Culture Lysis Reagent (Promega #E153A) according to company’s instructions in 96-well opaque Optiplates (Nunc). Luciferase activity was measured using 1420 Victor3 Multilabel Counter (PerkinElmer) with 10 second per well reading time. For SEAP encoding PsV, assays were evaluated using the Ziva Ultra SEAP Plus Kit (Jaden BioScience) according modified company’s instructions. After 48-hour incubation, plates were shaken for a minute to gain a homogenous supernatant. Supernatants were centrifuged for 5 minutes at 1700x*g* and 5μL transferred onto a 96-well Optiplate (Nunc) containing 75μL SEAP sample preparation solution. Plates were shaken again and incubated at 65–68°C for 50 minutes. Following inactivation of endogenous SEAP, 25μL of substrate were added using the Victor3 dispenser and plates incubated in the dark for 30 minutes prior to reading luminescence for 0.3 seconds per well. For CHOΔfurin conditioned medium, 1x10^6^ cells were grown in 17mL media and supernatants harvested after incubation at 37°C for 4 days.

### Elispot

Groups of C57BL/6 mice (n = 3) were immunized twice sub-cutaneously (day 0 and 10) using either 2μg HPV18L1–45RG1 VLP, HPV18L1 VLP, or 200μl PBS and sacrificed on day 20. Spleens were collected, dissociated into single cell suspension, erythrocytes were lysed (0.155M NH_4_Cl, 10mM KHCO_3_, 0.1M EDTA; pH 7.4), and splenocytes washed with PBS and resuspended in complete medium (RPMI1640, 10% FCS, 1X Pen-Strep, 1X NEAA, Sodium-pyruvate, 50μM β-mercaptoethanol). Elispot plates (Millipore Multiscreen IP filter plates; eBioscience Mouse anti-IFNγ ELISPOT Ready Set Go Kit) were pre-wetted with ethanol, washed with coating buffer (PBS, 0.05% Tween 20) and coated with IFN-γ capture-antibody for 24h at 4°C. The next day, plates were washed and blocked with complete medium at 37°C during splenocyte preparation. Finally, cell suspensions of 10^5^, 5*10^5^ and 10^6 cells^/_ml_ (in triplicates) were plated onto Elispot plates in the presence of 2μg/ml final concentration of *S*. *aureus* enterotoxin A (SEA, Sigma Aldrich), or 5μM of either HPV45RG1 peptide, or HPV18L1 VLP, or a combination of HPV18L1 VLP + HPV45RG1 peptide. Serum (FCS) alone or scrambled peptide were used as negative controls, respectively.

Biotinylated anti-IFNγ antibodies were used as conjugate and plates incubated with streptavidin-HRP. IFNγ producing cell spots were developed using Dako AEC + High sensitivity Substrate Chromogen (Dako, K3469) and counted. Results are expressed as mean number of spots forming cells ± standard deviation (SD).

### *In vivo* vaginal pseudovirion challenge

Six to eight weeks-old female Balb/c mice were obtained from Charles River Laboratory (Germany). In vivo challenge experiments were performed according to Roberts et al. [34]. Briefly groups of mice were synchronized by s.c. injection of 3mg progesterone (Depocon; Pfizer), three days prior to passive immunization (20μl, intra-venous (i.v.)) with pre-immune-serum (n = 10) or immune sera raised against HPV18L1 wt VLP (n = 5) or chimeric HPV18L1–45RG1 VLP (n = 5; serum #2), or sera against the respective specific L1 VLP type (n = 5). One day later, 30μl of a 1:1 PsV-carboxymethylcellulose (CMC) mixture was vaginally installed using a positive displacement pipette (Gilson), following mechanical disruption of the cervico-vaginal mucosa using a cytobrush (Cooper Surgical Company; C0012). Three days later, infection was assessed by intravaginal installment of 20μl luciferin (7.5^mg^/_ml_; Promega) and imaging using a Xenogen IVIS50 system (Perkin Elmer). Data were analyzed with the Living image software program by drawing uniform regions of interest (ROI) around the luciferase emitting genital area of each mouse to determine the average radiance within the ROI. A group of mice (n = 5) was challenged with CMC only to determine background radiance. Luciferase activity was measured as p/s/cm2/sr (average radiance).

### Statistics

Statistical analysis was performed using Graph Pad Prism to evaluate p-values for the overall experiment (1-way ANOVA analysis) and to compare pre-immune versus HPV18L1–45RG1 VLP groups (unpaired, two-tailed t-test).

### Ethics Statement

Animal studies were approved by the ethics committee of the Medical University of Vienna and the Austrian Federal Ministry of Science and Research (BMWF-66.009/0173–1l/3b/2011) and animal care was in accordance to the guidelines of the Veterinary University of Vienna.

## Results

**Fig 1:**
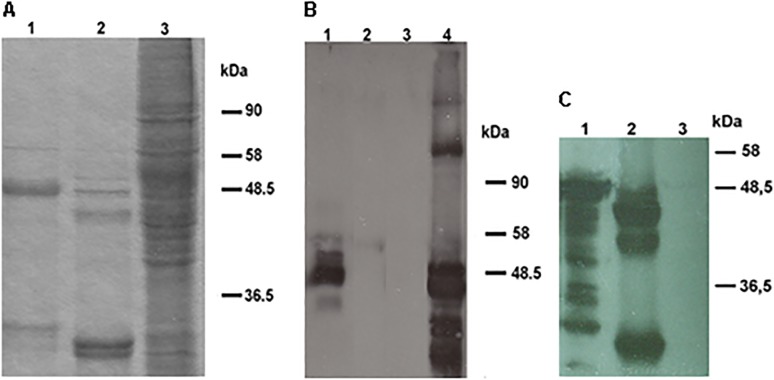
SDS-PAGE-Coomassie staining and Western Blot of HPV18L1–45RG1 VLP. Purified and dialyzed 18L1–45RG1 VLP (lane 1), HPV18 wt L1 VLP (lane 2) or crude Sf9 lysate (lane 3) were separated by SDS-PAGE followed by Coomassie staining (A). The L1-RG1 fusion protein migrated at a molecular weight of about 50kDa, slightly slower than wt HPV18 L1, for which smaller degradation products are also visible. Insertion of the RG1 peptide into the L1 protein and its antigenicity were verified by Western Blot using an anti-HPV16 L2 aa 11–200 serum (B), or Camvir-1 reacting to HPV18 L1 (C). For both Western Blots, HPV18L1–45RG1 fusion proteins show a molecular weight of about 50kDa (Fig. 1B lane 1; Fig. 1C lane 1), with smaller bands representing proteolytic degradation products. As controls HPV18 wt L1 VLP (Fig. 1B lane 2; 1C lane 2), HPV16L1L2 VLP (Fig. 1B lane 4) and Sf9 cells only (Fig. 1B lane 3; Fig. 1C lane 3) were used.

The 18L1–45RG1 chimeric gene was designed *in silico*, codon-optimized for mammalian expression, synthesized and cloned into a baculovirus transfer vector for recombination and expression in Sf9 insect cells. Recombinant baculoviruses were generated by established methods, isolated by plaque assay, high-titer viral supernatants were generated and used to infect Sf9 cells at high multiplicity of infection (MOI). Expression of the recombinant fusion protein was verified by SDS-PAGE and Coomassie staining (Fig. 1A), and antigenicity by Western Blot using an anti-HPV16 L2 aa 11–200 serum (Fig. 1B), or anti-L1 mAb Camvir-1 (Fig. 1C). The L1-RG1 chimeric fusion protein shows a full-length band at approximately 50kDa in both L1 and L2 Western Blots (Fig. 1B lane 1 and Fig. 1C lane 1), whereas additional faster migrating bands indicate proteolytic degradation products.

**Fig 2:**
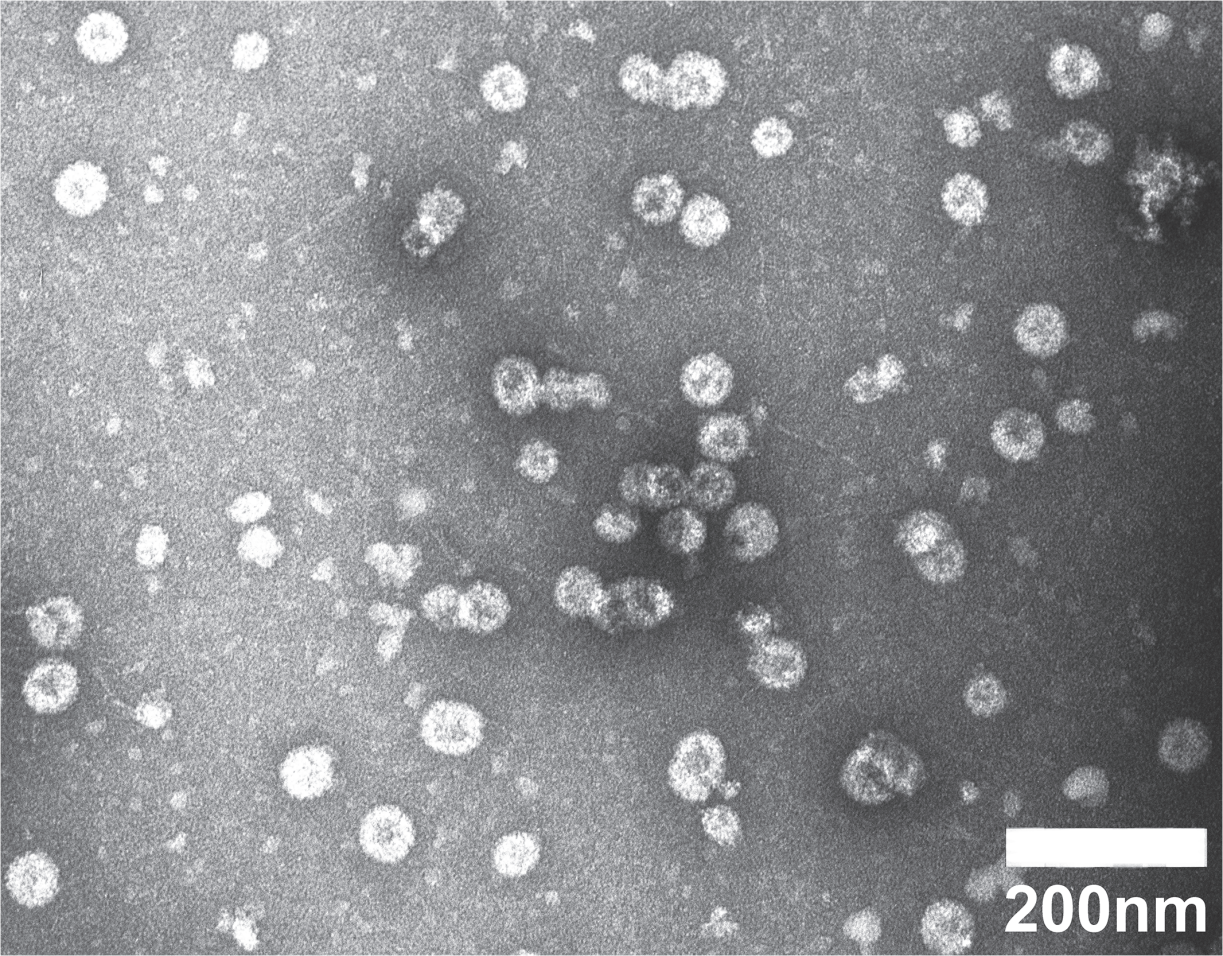
Transmission electron microscopy of chimeric HPV18L1–45RG1 VLP. VLP were gradient-purified, negatively stained and visualized at 30,000-fold magnification using a JEOL 1010 electron microscope. The size of the bar indicates 200nm.

Following purification of high molecular weight structures from baculovirus infected Sf9 cell lysates by density gradient centrifugation, preparations were negatively stained and visualized by TEM. Fig. 2 shows spherical particles with 50–60nm diameter, and also smaller and irregular particles, and considerable amounts of pentamers, indicating that despite insertion of the 20aa 45RG1 epitope into an L1 surface loop, the 18L1–45RG1 chimeric protein retains the ability to self-assemble into VLP that are predicted to display the L2 peptide repetitively (up to 360x) on the particle surface.

**Fig 3:**
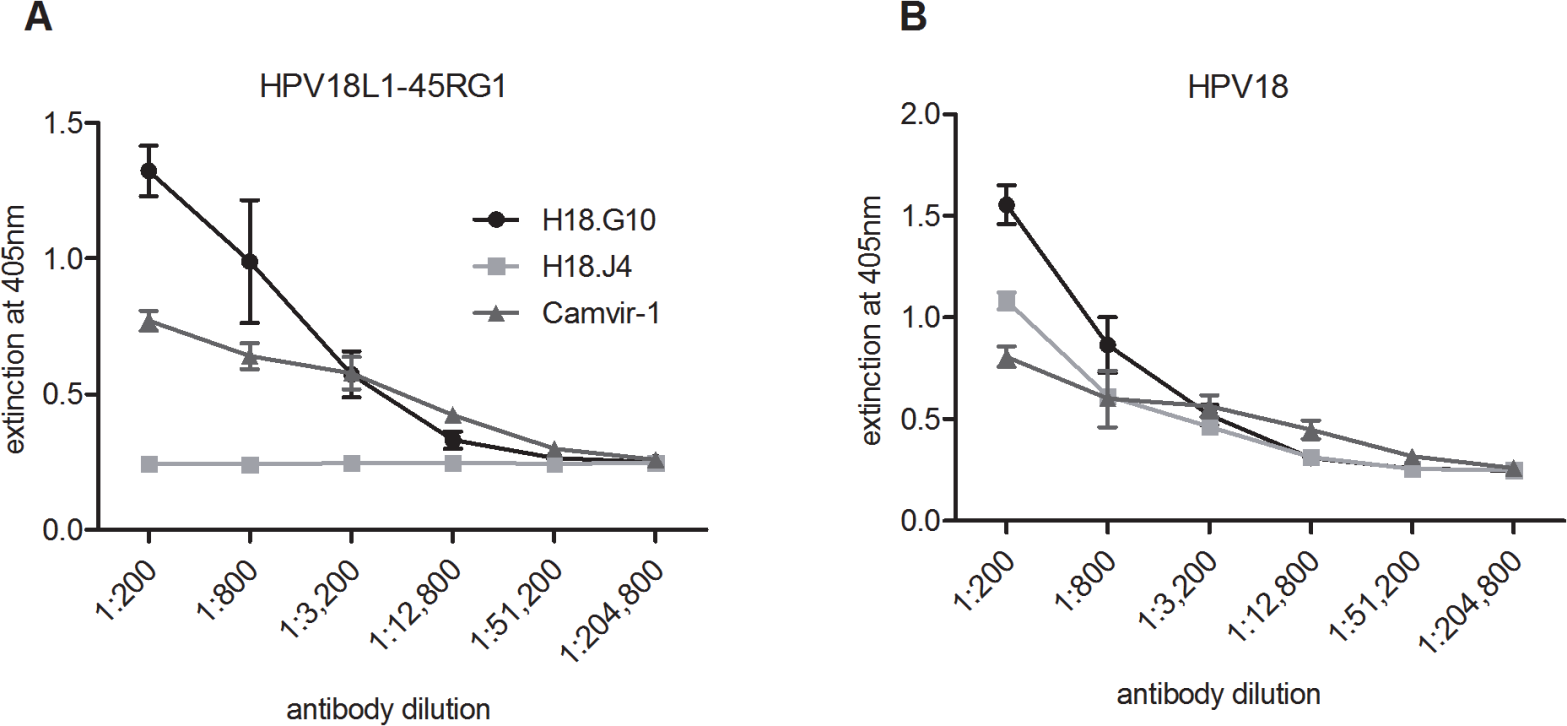
Characterization of chimeric HPV18L1–45RG1 by ELISA. Chimeric 18L1–45RG1 VLP (left) or wt HPV18L1 VLP (right) (50ng per well) were used as antigens and attached to 96 well plates under native conditions. ELISA was performed in triplicates with fourfold serial antibody endpoint dilutions ranging from 1:200–1:204,800 using HPV18-specific conformational-dependent neutralizing mAb H18.G10 and H18.J4, or non-neutralizing mAb Camvir-1 directed to a linear L1 epitope. Results indicate that RG1 epitope insertion into HPV18L1–45RG1 VLP disrupted (or sterically hindered) the recognition site of H18.J4, whereas the sites for H18.G10 and Camvir-1 remained intact. Data are shown as mean OD ± standard deviation (SD).

Antigenicity of chimeric VLP was verified and compared to wt HPV18L1 VLP by ELISA under non-denaturing conditions, using two HPV18-specific conformation-dependent mAb H18.G10 and H18.J4 [35], or Camvir-1 directed to a linear L1 epitope as control. The neutralizing mAb H18.G10 bound to both chimeric (Fig. 3A) and wt (Fig. 3B) VLP, suggesting that the native structure of the VLP scaffold required for the conformational neutralizing L1 epitopes was (at least in part) retained. In contrast, mAb H18.J4 only bound to wt VLP, indicating that insertion of the RG1 epitope had either disrupted the H18.J4 recognition site, or sterically hindered mAb binding. As expected, Camvir-1 detected both proteins expressing the linear L1 epitope.

**Fig 4:**
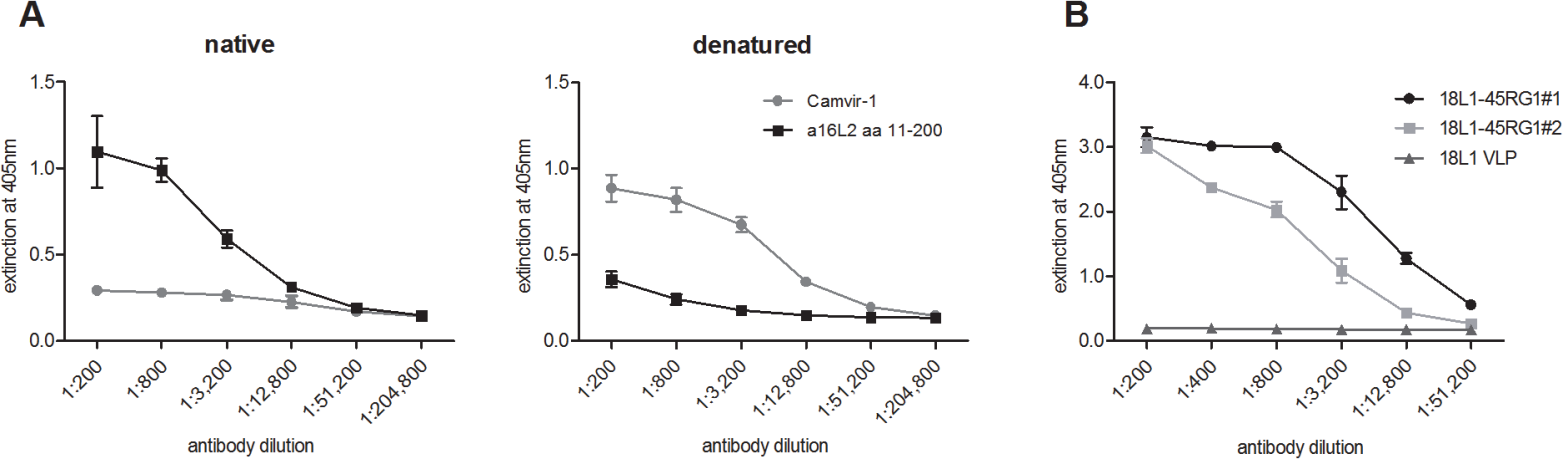
ELISA to characterize VLP surface-display of RG1-peptide and to detect antibodies to RG1 induced by 18L1–45RG1 VLP vaccination. (A) Native or denatured HPV18L1–45RG1 VLP were attached to 96-well ELISA plate and contacted with serial dilutions of anti-HPV16 L2 polyclonal serum recognizing the RG1 epitope. The anti-L2 serum recognized both native and denatured chimeric VLP, indicating RG1 display on the surface of VLP. As a control, binding of mAb Camvir-1 was assessed, which is directed to a linear L1 epitope hidden in the assembled protein. (B) Biotinylated peptides representing HPV45 RG1 were attached to 96-well Streptavidin plates. ELISA was performed in triplicates using either immune sera raised to HPV18L1–45RG1 VLP or HPV18L1 VLP, or pre-immune sera, with dilutions ranging from 1:200 to 1:51,200. HPV18L1–45RG1 immune sera, but not HPV18L1 VLP sera or pre-immune sera, bound to the RG1 peptide at titers of 12,800 to 51,200 (serum #2 and #1, respectively). Data are shown as mean OD ± standard deviation (SD) and titers reported as mean values 3 SD above background signals (pre-immune values).

To assess whether the RG1 epitope is exposed on the surface of chimeric RG1 VLP, ELISA experiments were performed with a rabbit polyclonal antiserum (raised against HPV16L2 aa 11–200) cross-reacting with HPV18 RG1, and using native or denatured HPV18L1–45RG1 VLP as ELISA antigens (Fig. 4A). The anti-RG1 serum recognized both native particles and denatured protein, indicating exposure of the RG1 epitope on the VLP surface. Binding was stronger to native VLP than to denatured protein, indicating some conformation of the RG1 epitope. As a control, binding of mAb Camvir-1, which is directed to a linear L1 epitope hidden in the assembled structure, was strongly increased for denatured protein.

To analyze immunogenicity of chimeric VLP, two NZW rabbits were immunized 5 times using alum-MPL adjuvant and immune sera analyzed 2 weeks after the final boost by ELISA, using HPV45 RG1 (Fig. 4B) biotinylated peptide as ELISA antigen. As shown in Fig. 4B, both immune sera (#1 and #2) raised against HPV18L1–45RG1 bind the RG1 peptide with titers ranging from 12,800–51,200, indicating immunogenic presentation of the cross-neutralizing RG1 epitope by the VLP vaccine.

### HPV18L1–45RG1 VLP immune sera (cross-)neutralize α7 HPV types *in vitro*

A PsV-based in vitro neutralization assay was used to analyze immune sera for (cross-)neutralizing antibodies against five hr α7 HPV types HPV18, 39, 45, 59 and 68, lr α7 HPV70, and hr α9 HPV16, 31. Type-specific immune sera raised against PsV or VLP of the respective types were used as controls. As shown in Table 1, vaccination with 18L1–45RG1 VLP induced high-titer neutralizing antisera against homologous type HPV18 PsV. In addition, cross- neutralization against related α7 types HPV39, 45 and 70 (titers ranging from 25–10,000), and very low titers against HPV68 (0–25) were detected, whereas no detectable titers were seen for the α7 hr type HPV59 and α9 HPV16, 31.

**Table 1:** In vitro cross-neutralization of α7 mucosal hr and lr HPV by antisera raised against 18L1–45RG1 VLP using L1-based pseudovirion neutralization assays.

		18L1–45RG1 VLP sera		type-specific VLP/PsV serum	HPV18L1 VLP serum
		#1	#2		
Pseudovirion		Neutralization Titer			
HPV16	hr α9	0	0	100,000	0
HPV31	hr α9	0	0	100,000	0
HPV18	hr α7	10,000	10,000	1,000,000	1,000,000
HPV39	hr α7	(25)	100	100,000	0
HPV45	hr α7	100	100	100,000	1,000
HPV59	hr α7	0	0	100,000	0
HPV68	hr α7	0	(25)	100,000	0
HPV70	lr α7	100	100	1,000,000	0

Two NZW rabbits were vaccinated with 18L1–45RG VLP and immune sera isolated. In addition, one rabbit each was immunized with wt L1 VLP or PsV of indicated types HPV16, 31, 18, 39, 45, 59, 68 and 70, to generate the respective type-specific immune sera. Pseudovirion-based neutralization assays were performed in duplicates and sera tested by serial dilution ranging from 1:25 to 1:100,000, or 1:100–1:1,000,000 for type-specific control sera, or 1:25–1:10,000,000 for the HPV18L1 wt control, respectively. Pre-immune sera analyzed did not show any (cross-)neutralizing activity (data not shown). Neutralization titers are reported as the reciprocals of the highest serum dilution causing 50% SEAP activity reduction compared to pre-immune sera.

Anti-L2 neutralizing antibody responses are more difficult to measure by L1-based neutralization assays, thus Day et al have recently developed an L2-based PsV assay that is >100-fold more sensitive to detect L2-specific neutralizing antibodies [33]. Using this novel assay analysis of immune sera against 18L1–45RG1 VLP revealed improved titers against the α7 types HPV39 (100), 45 (1,000), 68 (0–100) and 70 (100–1,000), and very low titers against HPV59 (0–25), for at least one of the two sera (Table 2, shown in bold). Neutralization titers against homologous type HPV18 were similar in both assays (10,000; mostly by antibodies directed to L1 epitopes), and were non-detectable for HPV16 and 31, as expected. Control serum raised against HPV18 L1 VLP showed very high titer specific for the homologous type HPV18, and cross-neutralizing titers of 1,000 to the closely related HPV45.

**Table 2:** In vitro cross-neutralization of α7 mucosal hr and lr HPV by antisera raised against 18L1–45RG1 VLP using L2-based pseudovirion neutralization assay.

18L1–45RG1 VLP sera				type-specific VLP/PsV serum	HPV18L1 VLP serum
		#1	#2		
Pseudovirion		Neutralization Titer			
HPV16	hr α9	0	0	10,000	0
HPV31	hr α9	0	0	10,000	0
HPV18	hr α7	10,000	10,000	100,000	100,000
HPV39	hr α7	**100**	100	1,000,000	0
HPV45	hr α7	**1,000**	**1,000**	1,000,000	1,000
HPV59	hr α7	(25)	0	1,000,000	0
HPV68	hr α7	0	**100**	1,000,000	0
HPV70	lr α7	**1,000**	100	1,000,000	0

The same immune sera as shown in Table 1 were used in this L2-based pseudovirion assay that more closely mimics the in vivo situation. Assays were performed in triplicates using serial serum dilutions ranging from 1:25 to 1:100,000, or 1:100 to 1:1,000,000 for type-specific controls, or 1:25–1:100,000 for the HPV18L1 VLP serum, respectively. Pre-immune rabbit sera were all non-neutralizing (data not shown). Improved cross-neutralizing titers as compared to those observed by L1-based assays (Table 1) are shown in bold. Neutralization titers are reported as the reciprocals of the highest serum dilution causing 50% SEAP/luciferase activity reduction compared to pre-immune sera.

### 18L1–45RG1 VLP vaccination induces a Th1 cellular immune response

Prophylactic HPV vaccination-induced humoral immune responses, in particular neutralizing antibodies, are thought to be the main effectors of protection, although the exact immune correlate and minimum antibody levels required for successful protection are not known. Th1 immune responses play an important role in infections with intracellular microbes and are induced in natural HPV infection mediating viral clearance and providing B cell help.

**Fig 5:**
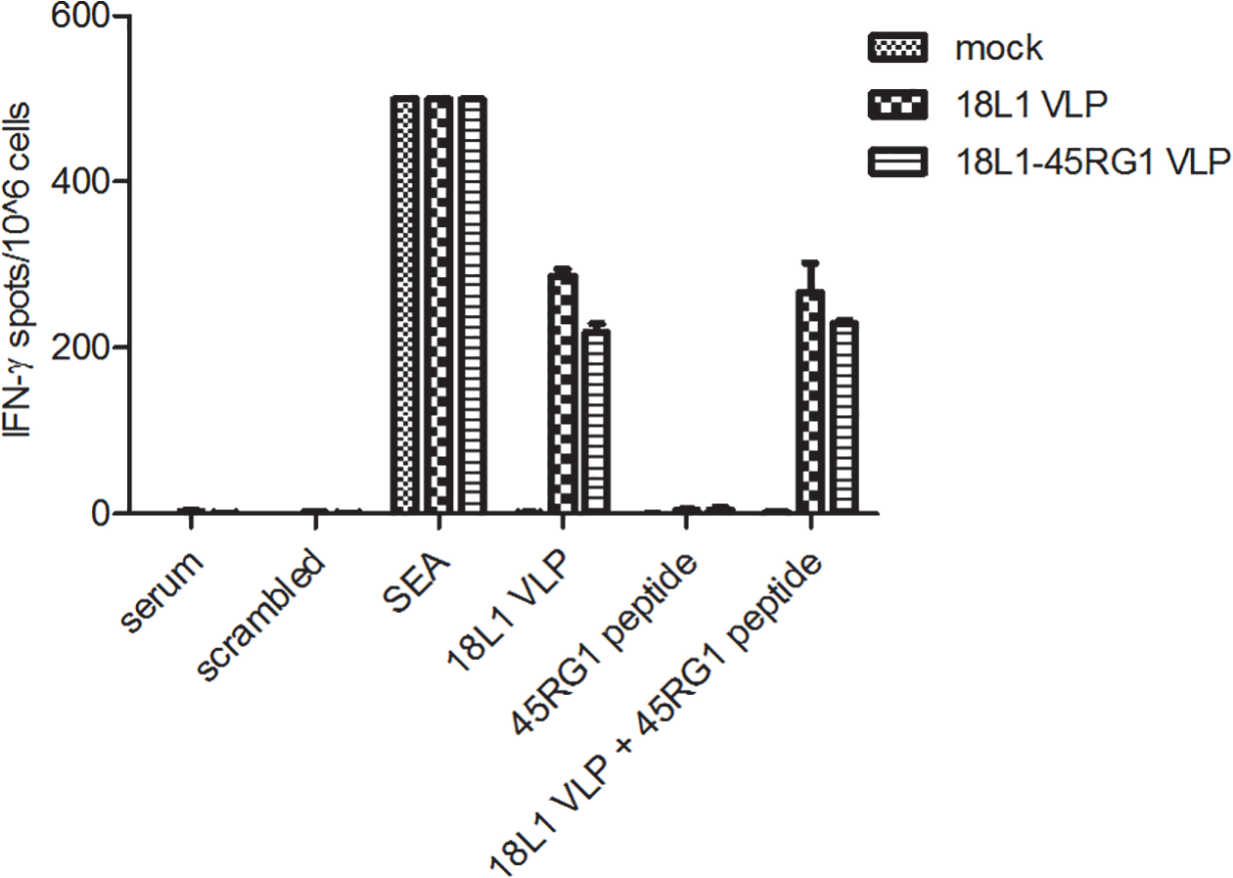
Th1 cell—mediated immune response induced by HPV18L1–45RG1 VLP vaccination by measuring IFN-γ by Elispot. Groups of C57BL/6 mice (n = 3) were s.c. immunized twice (day 0 and 10) with 2μg of either wt HPV18L1 VLP, chimeric HPV18L1–45RG1 VLP, or PBS as mock control. On day 20 spleens were harvested, groups pooled and splenocytes isolated. 10^6^ splenocytes were plated onto Elispot plates, and stimulated with either wt HPV18L1 VLP, or HPV45 RG1 synthetic peptide, or a combination of both. The graphs show that HPV18L1 VLP, but not the RG1 peptide, induced IFN-γ production in splenocytes of HPV18L1–45RG1 or wt HPV18L1 VLP pre-sensitized mice. Shown are mean values ± SD of triplicate cultures.

To analyze if chimeric VLP vaccination induces a cellular immune response, C57BL/6 mice were immunized twice (days 0, 10) with 18L1–45RG1 VLP or wt 18L1 VLP, and splenocytes were isolated (day 20) and analyzed by ELISPOT. A group of mice immunized with PBS was used as mock control. Due to the lack of a described HPV18L1 CTL epitope, HPV18L1 VLP were employed as stimulus. As shown in Fig. 5, splenocytes from mice immunized with 18L1–45RG1 or wt 18L1 VLP showed IFN-γ induction when stimulated with HPV18L1 VLP, but not with HPV45 RG1 peptide alone. Positive (SEA) and negative controls (serum and scrambled peptide) showed the expected results.

### Passive transfer of rabbit antisera raised against 18L1–45RG1 VLP protects mice against vaginal challenge with PsV of hr mucosal α7 HPV types

**Fig 6:**
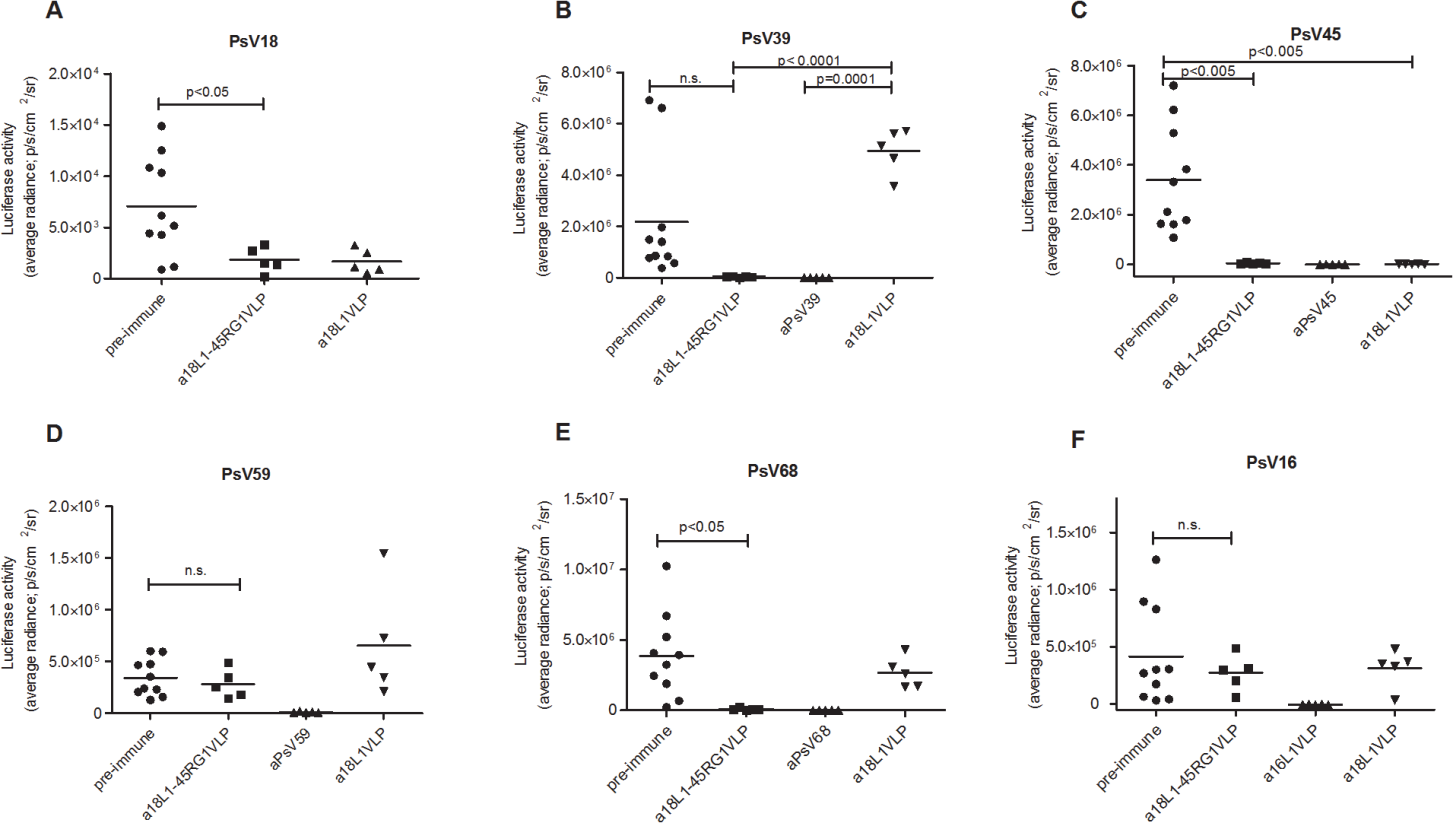
HPV18L1-45RG1 VLP vaccine efficacy against experimental genital challenge with hr α7 HPV18, 39, 45, 59, 68 pseudovirions in mice. Progesterone synchronized groups of mice were passively immunized by intravenous transfer of 20μl of pre-immune or immune sera raised to HPV18L1–45RG1 VLP, 18L1 wt VLP, or a type-specific immune serum. 24 hours later, the vaginal epithelium was mechanically disrupted followed by intravaginal installation of indicated pseudovirions (HPV18, 39, 45, 59, 68 or 16) enclosing a luciferase gene. Three days later infection was detected by a bioluminescence imager (IVIS). Rabbit antiserum to HPV18L1–45RG1 VLP conferred levels of protection similar to type specific L1 sera for HPV18, 39, 45 and 68 (p-values of 0.0151; 0.0003; 0.0001 and 0.0038, respectively, using One-way ANOVA), but not to HPV59 or HPV16 PsV (p-values of 0.0099 and 0.1114). In contrast, HPV18L1 VLP sera conferred mostly type-restricted protection to HPV18 and cross-protection against HPV45 only (t-test p-values of 0.0311 and 0.0044). Luciferase activity was measured as p/s/cm2/sr (average radiance) and results are shown after subtraction of background luminescence (unvaccinated mice challenged with CMC only). P-values for significant differences between pre-immune versus HPV18L1–45RG1 VLP groups (t-test) are shown or indicated not significant (n.s.).

A murine vaginal challenge model (adapted from [34]) was used to evaluate in vivo efficacy of 18L1–45RG1 VLP vaccination. In accordance with in vitro neutralization data, passive i.v. transfer of 20μl immune serum raised against chimeric VLP (rabbit #2), with an estimated 1:50 dilution in the mouse circulation, afforded complete protection to mice against experimental vaginal challenge with PsV from the cognate mucosal hr type HPV18 (Fig. 6A), reducing the signals to background level. Efficacy of protection was similar to that afforded by antiserum to wt 18L1 VLP, whereas pre-immune serum had no protective effect. In addition, anti-18L1–45RG1 sera provided highly efficient cross-protection against challenge with non-cognate α7 types HPV39, 45 and 68 (Fig. 6B, C, and E), but not against HPV59 (Fig. 6D). In contrast, sera to wt HPV18L1 VLP only provided restricted protection against challenge with cognate HPV18 and the closely related type HPV45. As expected, immune sera to chimeric VLP or wt HPV18L1 VLP did not protect against HPV16 challenge (Fig. 6F).

Comparisons of pre-immune and 18L1–45RG1 VLP vaccine groups alone show significant protection against challenge with pseudovirions of types HPV18, 45 and 68 (t-test p-values of 0.0353, 0.0047 and 0.0163, respectively) (Fig. 6A, C, E), but not for HPV39 (t-test p–value of 0.0805) (Fig. 6B) and, as expected from in vitro result, not for HPV59 and HPV16 (t-test p-values of 0.5185 and 0.4852, respectively) (Fig. 6D, F). Comparison of pre-immune and anti-HPV39 type-specific groups also did not reach significance (p-value of 0.0749) (Fig. 6B), mostly because HPV39 PsV, for some reason, did not effectively infect the pre-immune group of mice. In contrast, PsV type 39 did infect the HPV18L1 VLP serum group more successfully and when comparing this group as reference to the HPV18L1–45RG1 serum group or the anti-HPV39 immunized group, statistical significance is reached (p-value of both <0.0001) (Fig. 6B).

## Discussion

Human papillomavirus types of species α7 comprise the second most important group of HPV that include mucosal hr types HPV18, 45, 39, 59 and 68, as well as lr type HPV70. Following HPV16, HPV18 is the second most important hr type causing 15% of CxC cases worldwide. Together, mucosal α9 HPV (with 7 hr types including HPV16), and α7 HPV cause almost all CxC (and HPV-associated non-cervical anogenital or oropharyngeal cancers). Further, α7 HPV types are overrepresented in AC of the endocervix, which are under-diagnosed by conventional PAP smears. Although cervical screening programs have led to a dramatic decline in the incidence of SCC, the incidence of cervical AC has remained stable or even increased in many countries [20,26,36–38].

Rising incidences of glandular AC may be due to better awareness of cytologists to recognize the precursor lesion AC in situ (AIS), as there is a parallel decline of carcinomas of “unspecified” histology and SCC cases. Also, recognition and diagnosis greatly depends on the sensitivity of technology and screening methods used [39]. By conventional PAP screen, cells are sampled mainly from the more accessible transformation zone of the ectocervix, whereas AC precursor lesions often arise deep in the endocervical canal and may even be covered by normal or metaplastic endothelial cells. It has been shown that PAP smear detected not more than 60% of AC or AIS cases while HPV testing more accurately detected this cancer with sensitivity of about 90% [40–42].

Given prophylactically, the licensed HPV vaccines are highly efficient against the two or four HPV types included (HPV6, 11, and/or 16, 18), yet show very restricted cross-protection against hr HPV31, 33 (closely related to HPV16), and HPV45 (closely related to HPV18) [43]. A nonavalent vaccine (V503, Merck) that contains 5 L1 VLP of types HPV31, 33, 45, 52 and 58 in addition to the original quadrivalent formulation (HPV16, 18, 6, 11, Gardasil), has been very effective in clinical studies and submitted for licensure to the FDA-US. However, V503 does not reduce complexity and probably high costs of current HPV vaccines, and it does not contain VLP of α7 hr HPV types HPV39, 59 and 68 (which together are responsible for ca. 2.6% of all CxC cases). Hence, there is still a need for advanced next generation HPV vaccines that broadly target oncogenic HPV types beyond HPV16 and HPV18, and at reduced costs to protect women particularly in developing countries suffering most from CxC.

HPV vaccines based on minor capsid protein L2 are a promising cost-effective alternative approach to current L1-VLP based vaccines. The L2 proteins of papillomaviruses are highly conserved and contain cross-neutralization epitopes, predominantly in the N-terminal aa 11–200 [13,14,44], that form the basis of several experimental second-generation HPV vaccines [11,16]. A multi-type L2 fusion protein with aa 11–88 from five HPV types, or aa 17–36 from 22 HPV types, can induce robust cross-neutralizing antibody titers [6,15]. To increase immunogenicity, different HPV16 L2 epitopes have been inserted into various positions of the L1 protein of HPV16 [45,46]. Further, L2 epitopes have been inserted into and displayed by VLP of papillomaviruses or bacteriophages [9] or bacterial thioredoxin scaffolds [47] and shown to induce cross-neutralizing antibodies. The RG1 epitope of HPV16 L2 (aa 17–36) appears particularly interesting, as it is conserved among many HPV types and vaccinations induce a broad cross-protective immunity against numerous HPV and even animal papillomavirus types [5,47,48]. Similarly, a single chimeric L1-L2 VLP can target a broad spectrum of heterologous HPV types [17,18].

Assembly into VLP or pentamers is an important aspect for HPV vaccine efficacy, as both VLP and pentamers have been shown to be strongly immunogenic as compared to non-assembled proteins or peptides. Analysis by TEM demonstrates that, despite insertion of the HPV45 RG1 epitope into the DE surface loop of HPV18L1, the chimeric protein very efficiently assembles into complete VLP, smaller particles, or pentamers (Fig. 2). Importantly, as shown by ELISA using native VLP as antigen, the binding epitope of a conformation-dependent neutralizing antibody to HPV18 is preserved (Fig. 3A), and the RG1 epitope displayed (Fig. 4A, B). Immunogenicity of RG1-VLP was analyzed by vaccination of NZW rabbits; induced immune sera showed limited in vitro cross-neutralization of α7 hr types as detected by L1-based PsV assay, the current standard to detect neutralizing antibodies and a correlate for vaccine efficacy (Table 1). However, a recently developed L2-based PsV neutralization assay has demonstrated increased sensitivity to detect anti-L2 neutralizing antibodies. Accordingly, significantly higher cross-neutralization titers to HPV39, 45, 68 and 70 up to 10-fold were detectable (Table 2) as compared to L1-based assays (Table 1). The antibody titers induced by 18L1–45RG1 VLP are 10–100 fold lower against the homologous type HPV18, compared to HPV18L1 VLP-induced titers. This is probably because insertion of the RG1 epitope has either disrupted a neutralizing epitope of L1, or impairs access via steric hindrance, as shown by the abrogation of one neutralizing mAb binding (H18.J4; Fig. 3A). Nevertheless, binding of another neutralizing mAb is preserved (H18.G10, Fig. 3A), and induced neutralization titers to HPV18 of 10,000 are expected to provide robust and long-lasting protection, similar to our previous findings with chimeric HPV16L1–16RG1 VLP (Schellenbacher 2009, 2013).

A murine genital PsV challenge model is regarded gold-standard for preclinical evaluation of HPV prophylactic vaccine efficacy. Passive transfer of antisera to 18L1–45RG1 VLP afforded (cross-)protection against vaginal challenge of mice with PsV of oncogenic α7 types HPV18, 39, 45 and 68. The latter three types together account for around 8% of all CxC worldwide, and HPV45 in particular for about 6% of the cases [2,49]. Cross-protection was similar to protection conferred by the respective type-specific immune sera raised against homologous L1-VLP (Fig. 6), despite several logs difference in serum titers (Table 1). Cross-neutralization of HPV68 was barely identified by L1-based assays in vitro (titer of 25, Table 1), but detectable for one serum (titer of 100) by L2-based assay (Table 2). This observation was corroborated in vivo, as immune serum cross-protected against HPV68 PsV challenge (Fig. 6), indicating that the current L1-based in vitro PsV neutralization assay lacks sufficient sensitivity to detect low level anti-L2 antibody responses and thus may underestimate full vaccine potential [33].

Unexpectedly, chimeric HPV18L1–45RG1 VLP did not induce cross-neutralizing antibody titers or in vivo protection against the closely related HPV59 (causing 1% of CxC worldwide) despite aa sequence homology of 85% between the RG1 epitopes of HPV45 and HPV59 (Table 3). In contrast, cross-neutralization of lr HPV70 was detected, the RG1 epitope of which shows sequence homology of 85% to HPV45’s RG1 as well. The RG1 peptides of HPV68 and HPV59 differ by only one aa (L2 residue 24 serine (S) versus alanine (A)) (Table 3), which appears responsible for lack of cross-neutralization of HPV59.

**Table 3:** RG1 amino acid sequence homology.

type	RG1 aa sequence (position 16–35)	% aa sequence homology to HPV45
45	DLYRTCKQSGTCPPDVINKV	
39	DLYRTCKQSGTCPPDV**VD**KV	90%
59	DLY**K**TCKQ**A**GTCP**S**DVINKV	85%
68	DLY**K**TCKQSGTCP**S**DVINKV	90%
70	D**I**Y**K**TCKQSGTCPPDV**V**NKV	85%

The highly conserved RG1 peptide of α7 HPV45 L2 (aa residues 16–35) is shown in comparison to the homologous RG1 peptides of α7 HPV39, 59, 68, 70. Differences in aa are indicated in bold.

Vaccination with HPV18L1–45RG1 VLP induced antisera that protect against 4 of 5 analyzed mucosal oncogenic α7 HPV types (HPV18, 39, 45 and 68). Such L1-RG1 VLP could be combined with the previously described HPV16 version of this construct to reduce complexity of proposed 9-valent multivalent vaccine formulations and therefore could reduce costs, as compared to adding additional L1 VLP. In addition, multiple hr mucosal HPV with low prevalence (<1%) in CxC are unlikely to be included into later generation L1 VLP vaccines and thus even vaccinated women would need to continue PAP screening. However, a combined HPV16+18 version of the RG1-chimeric VLP might afford sufficiently broad protection against hr HPV as to eventually eliminate the need for cervical screening in vaccinated women. Recent data also points to the possibility that a single dose of Cervarix might afford a durable protection against HPV16/18 infection. Therefore, additional animal studies addressing the potential of a single dose HPV16+18 version of the RG1-chimeric VLP are warranted given the similarities of the two immunogens and their adjuvant.

In summary, L1-RG1 VLP offers a promising approach to develop an extended-spectrum HPV vaccine to decrease the worldwide CxC burden, directed in particular against HPV-associated AC for which PAP-screening has been less effective.
